# A Rare Cause of Childhood Nephrotic Syndrome: AA Amyloidosis in Epidermolysis Bullosa

**DOI:** 10.7759/cureus.85553

**Published:** 2025-06-08

**Authors:** Ruveyda Gulmez, Saliha Yilmaz, Seha Saygili, Ozge Hurdogan, Ayse Agbas, Esra Karabag Yilmaz, Gozde Apaydın Sever, Nur Canpolat

**Affiliations:** 1 Pediatric Nephrology, Goztepe Prof. Dr. Suleyman Yalcin City Hospital, Istanbul, TUR; 2 Pediatrics, Istanbul University-Cerrahpasa, Cerrahpasa Faculty of Medicine, Istanbul, TUR; 3 Pediatric Nephrology, Istanbul University-Cerrahpasa, Cerrahpasa Faculty of Medicine, Istanbul, TUR; 4 Pathology, Istanbul University School of Medicine, Istanbul, TUR; 5 Pediatric Infectious Diseases, Istanbul University-Cerrahpasa, Cerrahpasa Faculty of Medicine, Istanbul, TUR

**Keywords:** childhood nephrotic syndrome, children, recessive dystrophic epidermolysis bullosa (rdeb), secondary amyloidosis, treatment choices

## Abstract

Epidermolysis bullosa (EB) is a rare, heterogeneous, hereditary, chronic skin disorder with severe cutaneous and extracutaneous involvement. With the significant increase in survival of EB patients, kidney complications have become more common. Among the EB subtypes, recessive dystrophic epidermolysis bullosa (RDEB) is associated with the development of amyloidosis. Secondary amyloidosis affecting the kidneys in RDEB is fatal due to its rapid progression and difficulty in dialysis. Herein, we present the case of a six-year-old boy diagnosed with EB who was referred to our center due to nephrotic syndrome. A kidney biopsy revealed amyloidosis with positive Congo red staining and amyloid fibrils on electron microscopy. Despite undergoing hemodialysis, the patient died at home shortly afterward from an unknown cause. This case highlights the importance of a proactive approach in EB management, emphasizing disease and inflammation control to reduce the risk of amyloidosis and kidney failure.

## Introduction

Epidermolysis bullosa (EB) is a heterogeneous group of rare genetic disorders characterized by mechanical fragility of the skin and mucous membranes, resulting in blistering, ulceration, and impaired wound healing in response to minimal trauma. EB is traditionally classified into four major types: epidermolysis bullosa simplex (EBS), junctional EB (JEB), dystrophic EB (DEB), and Kindler syndrome, based on the ultrastructural level of skin cleavage and associated gene mutations [1]. Among these subtypes, recessive dystrophic EB (RDEB) is known to cause the most severe extracutaneous manifestations, including chronic inflammation, recurrent infections, nutritional deficiencies, mucosal involvement, and systemic organ damage [1].

Advancements in wound care, nutritional support, and infection management have contributed to increased life expectancy in patients with severe forms of EB. As a consequence, long-term complications such as renal involvement have become more apparent. Kidney manifestations reported in EB include IgA nephropathy, post-infectious glomerulonephritis, hereditary nephritis, obstructive uropathy, and, rarely, secondary (AA) amyloidosis, the latter being the most serious form due to its poor prognosis and limited treatment options [2-8].

AA amyloidosis occurs as a result of prolonged elevation of serum amyloid A (SAA) protein, an acute-phase reactant, in the context of chronic inflammation. In RDEB, persistent skin wounds, recurrent sepsis, and uncontrolled inflammation can lead to continuous SAA overproduction, promoting systemic amyloid fibril deposition, especially in the kidneys [2]. In fact, renal involvement is reported in up to 80% of AA amyloidosis cases, typically manifesting as nephrotic syndrome or progressive chronic kidney disease [2]. Although systemic amyloidosis is well described in adult EB populations, pediatric cases remain rare, and nephrotic syndrome as the first sign of EB-related amyloidosis is exceedingly uncommon [9].

Several reports have proposed early colchicine therapy as a potential strategy to delay or mitigate renal involvement in AA amyloidosis, based on its established efficacy in familial Mediterranean fever (FMF) [10]. More recently, interleukin-targeting biologics such as anakinra (anti-IL-1) and tocilizumab (anti-IL-6) have shown encouraging results in systemic juvenile idiopathic arthritis (SJIA)-associated amyloidosis and other autoinflammatory diseases, although no studies to date have investigated their use in EB-related amyloidosis [11,12].

In this report, we present the case of a six-year-old child with RDEB whose nephrotic syndrome was the initial manifestation of biopsy-proven renal AA amyloidosis. The case underscores the importance of early recognition, regular screening for proteinuria, and consideration of anti-inflammatory interventions to prevent irreversible kidney damage in children with severe EB.

This article was previously presented as a poster at the 7th Young Pediatricians Congress on December 3, 2022.

## Case presentation

A six-year-old boy, the first child of the first-cousin parents, with a known diagnosis of EB, was referred to our center for generalized edema prior to syndactyly surgery. On admission, despite anasarca edema, he appeared to be cachectic (weight 18 kg (-1.84 SDS), height 115 cm (-1.24 SDS)). The physical examination revealed normal blood pressure (98/65 mmHg, 50-75th percentile for his height), a 2/6 systolic murmur, and extensive skin lesions, including multiple fragile blisters, intact bullae, large, eroded areas, and deep atrophic scars. His hands and feet exhibited contractures and pseudo-syndactyly, accompanied by sparse, dry hair and nail loss.

Laboratory tests showed anemia and thrombocytosis, increased inflammatory markers (erythrocyte sedimentation rate (ESR) and C-reactive protein (CRP)), increased serum urea and creatinine, hypoalbuminemia, and massive proteinuria. Serum electrolytes, blood gas, and complement levels (C3 and C4) were within normal limits. The detailed laboratory parameters are summarized in Table 1.

**Table 1:** Laboratory parameters on admission PLT: Platelet count, ESR: Erythrocyte sedimentation rate, CRP: C-reactive protein, C3: Complement component 3, C4: Complement component 4

Parameter	Value	Reference Range
Hemoglobin	8.6 g/dL	11.5–15.5 g/dL
PLT	503 × 10^3^/μL	150–400 × 10^3^/μL
ESR	55 mm/h	<20 mm/h
CRP	61 mg/L	<5 mg/L
Urea	74 mg/dL	10–50 mg/dL
Creatinine	1.0 mg/dL	0.3–0.7 mg/dL
Sodium	140 mEq/L	135–145 mEq/L
Potassium	4.1 mEq/L	3.5–5.0 mEq/L
Chloride	113 mEq/L	98–108 mEq/L
Calcium	5.1 mg/dL	8.4–10.8 mg/dL
Phosphate	7.5 mg/dL	3.5–6 mg/dL
Total protein	6.8 g/dL	6-8 g/dL
Albumin	0.68 g/dL	3.5–5.5 g/dL
pH	7.40	7.35-7.45
HCO_3_	19.7 mmol/L	21-28 mmol/L
Spot urine protein/creatinine	50 mg/mg	<0.2 mg/mg
C3	0.96 g/L	0.9–1.8 g/L
C4	0.27 g/L	0.1–0.4 g/L

**Figure 1:**
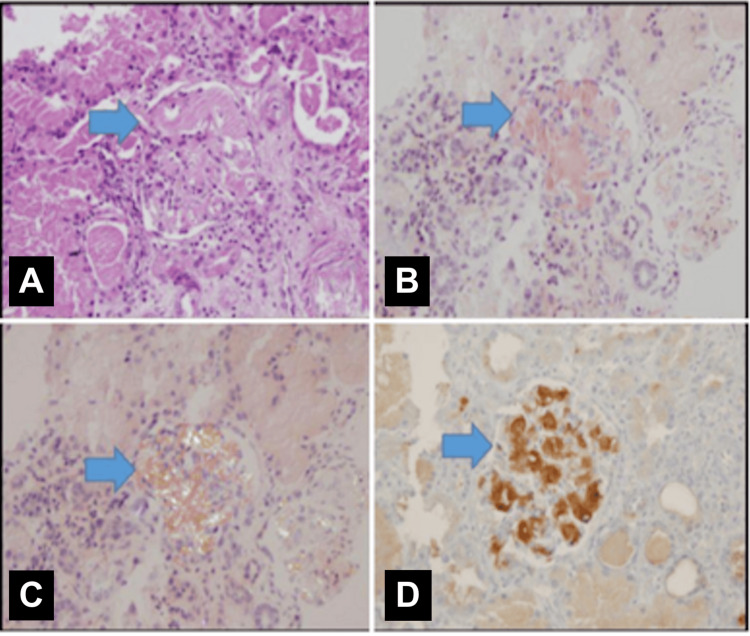
Histopathological and immunohistochemical evidence of glomerular AA amyloid deposition in a kidney biopsy Renal biopsy findings. (A) Eosinophilic substance accumulation in the mesangial region and basement membrane in the glomeruli (hematoxylin & eosin, ×400); (B) positiveness with Congo stain in the accumulation of eosinophilic substance in the glomeruli (Congo, ×400); (C) apple green birefringence under polarized light with Congo dye in the glomeruli (Congo, polarized light, ×400); (D) immunohistochemical reaction with amyloid AA antibodies in glomeruli (anti-amyloid AA, ×400).

A chest X-ray performed at admission was unremarkable; however, during the oliguric phase, it revealed findings consistent with volume overload. The echocardiography report was unavailable in the medical records. The kidney ultrasound showed bilaterally enlarged kidneys (right and left kidney length 118 and 120 mm, respectively, both >100th percentile for age) with increased echogenicity and loss of corticomedullary differentiation. A kidney biopsy confirmed amyloidosis, with positive Congo red staining observed under polarized light and electron microscopy (Figure 1).

Colchicine treatment was initiated; however, kidney function rapidly deteriorated, accompanied by a decrease in urine output. One month after admission, the patient became anuric and required dialysis to be initiated. No other organ involvement related to amyloidosis was identified. While undergoing intermittent hemodialysis, he died at home of an unknown cause three months after his first hospital admission.

## Discussion

AA amyloidosis is a recognized, though rare, complication of chronic inflammatory conditions such as EB, more commonly reported in adult patients. This case represents one of the youngest pediatric patients with RDEB to develop secondary amyloidosis, with a notably rapid progression to end-stage kidney disease (ESKD) and subsequent death within a short period.

In AA amyloidosis, sustained inflammation or recurrent infections lead to elevated SAA protein levels, resulting in progressive amyloid deposition in various organs. The kidneys are affected in approximately 80% of patients with AA amyloidosis [2]. Amyloid deposition in the glomeruli may cause nephrotic syndrome, whereas involvement of the tubulointerstitial or vascular compartments may result in renal impairment with minimal or no proteinuria [1,4]. While RDEB is caused by mutations in the *COL7A1* gene, genetic predispositions beyond the primary mutation, such as polymorphisms in genes regulating inflammation and amyloid clearance, may also influence susceptibility to AA amyloidosis. Although such associations have been described in other autoinflammatory diseases, we were unable to evaluate these factors in our patient due to a lack of genetic testing and regular follow-up.

Amyloidosis often presents with nonspecific and delayed clinical signs, which complicates timely diagnosis. Retrospective evaluation of our patient's electronic medical records revealed that proteinuria and hypoalbuminemia had already been detected one month prior to admission. In conditions such as FMF, the progression from proteinuria to ESKD typically takes three to four years [13]. In contrast, reported EB-related cases describe variable progression, ranging from 2 to 10 years [13]. Alarmingly, some cases progressed from initial proteinuria to dialysis or death within 15-20 months [14]. In our case, disease progression was particularly aggressive, with ESKD developing within two months of diagnosis and death occurring three months after dialysis initiation. Kidney replacement therapy presents unique challenges in EB patients due to severe skin fragility. Catheter insertion and maintenance can lead to mechanical trauma and increase the risk of infections. Despite this, our patient did not experience catheter-related complications during dialysis.

Colchicine remains the mainstay of therapy in AA amyloidosis, especially when initiated early. In FMF, it has demonstrated efficacy in delaying or preventing renal involvement. While limited, emerging evidence also suggests a potential benefit in EB-related amyloidosis. In a case series by Kaneko et al. [10], adult patients who received colchicine had more stable renal function compared to those who did not. Additionally, a pediatric report involving two siblings noted a decrease in serum SAA levels following colchicine treatment, despite ongoing proteinuria. Unfortunately, in our case, colchicine was started at a late stage, likely limiting its therapeutic effect.

Beyond colchicine, biologic agents such as anakinra (IL-1 receptor antagonist) and tocilizumab (IL-6 inhibitor) have shown promise in treating secondary amyloidosis in systemic autoinflammatory diseases, including SJIA [11,15]. A review of 13 pediatric SJIA patients treated with tocilizumab reported clinical and biochemical improvement in nearly half [16]. Similarly, anakinra has been associated with reduced proteinuria and symptomatic improvement, although renal histology findings remained unchanged in some cases [12]. However, no studies to date have explored the use of biologics in EB-associated AA amyloidosis. Given the high mortality, rapid disease progression, and limited treatment success in advanced cases, early diagnosis and timely intervention are crucial. There is a pressing need for clinical studies and the development of standardized treatment guidelines to evaluate the role of biologic therapies, potentially in combination with colchicine, in improving outcomes in EB-related amyloidosis.

## Conclusions

The prognosis for patients with EB and secondary amyloidosis is generally poor, especially when amyloid deposition affects vital organs like the kidneys. Early diagnosis and intervention, particularly with colchicine, are crucial, but the effectiveness of current therapies remains limited in advanced cases. Future treatments could potentially include targeted therapies aimed at reducing amyloid fibril production or enhancing the clearance of amyloid deposits. The role of stem cell therapy, gene editing, and biologic agents in the treatment of EB-related amyloidosis is an area of ongoing research. Early screening, timely intervention, and research into novel therapeutic options are essential to improve survival in this life-threatening condition.
